# Targeting of immunosuppressive myeloid cells from glioblastoma patients by modulation of size and surface charge of lipid nanocapsules

**DOI:** 10.1186/s12951-020-00589-3

**Published:** 2020-02-17

**Authors:** Laura Pinton, Sara Magri, Elena Masetto, Marina Vettore, Ilaria Schibuola, Vincenzo Ingangi, Ilaria Marigo, Kevin Matha, Jean-Pierre Benoit, Alessandro Della Puppa, Vincenzo Bronte, Giovanna Lollo, Susanna Mandruzzato

**Affiliations:** 1 Veneto Institute of Oncology IOV–IRCCS, Padua, Italy; 2 Department of Surgery, Oncology and Gastroenterology, University of Padova, Via Gattamelata 64, 35128 Padua, Italy; 3 Pharmacy Department, Academic Hospital, 4 rue Larrey, Angers, France; 4 Micro et Nanomedecines Translationnelles, MINT, UNIV Angers, UMR INSERM 1066, UMR CNRS 6021, Angers, France; 5 Neurosurgery Unit, Azienda Ospedaliera di Padova, Padua, Italy; 6 Department of Medicine, Section of Immunology, University of Verona, Verona, Italy; 7 Univ Lyon, Université Claude Bernard Lyon 1, CNRS, LAGEPP UMR 5007, 69100 Villeurbanne, France; 8 Present Address: Department of NEUROFARBA, University Hospital of Careggi, University of Florence, Florence, Italy

**Keywords:** Myeloid cells, Lipid nanocapsules, Glioma, Myeloid derived suppressor cells, Immunosuppression

## Abstract

**Background:**

Myeloid derived suppressor cells (MDSCs) and tumor-associated macrophages (TAMs) are two of the major players involved in the inhibition of anti-tumor immune response in cancer patients, leading to poor prognosis. Selective targeting of myeloid cells has therefore become an attractive therapeutic strategy to relieve immunosuppression and, in this frame, we previously demonstrated that lipid nanocapsules (LNCs) loaded with lauroyl-modified gemcitabine efficiently target monocytic MDSCs in melanoma patients. In this study, we investigated the impact of the physico-chemical characteristics of LNCs, namely size and surface potential, towards immunosuppressive cell targeting. We exploited myeloid cells isolated from glioblastoma patients, which play a relevant role in the immunosuppression, to demonstrate that tailored nanosystems can target not only tumor cells but also tumor-promoting cells, thus constituting an efficient system that could be used to inhibit their function.

**Results:**

The incorporation of different LNC formulations with a size of 100 nm, carrying overall positive, neutral or negative charge, was evaluated on leukocytes and tumor-infiltrating cells freshly isolated from glioblastoma patients. We observed that the maximum LNC uptake was obtained in monocytes with neutral 100 nm LNCs, while positively charged 100 nm LNCs were more effective on macrophages and tumor cells, maintaining at low level the incorporation by T cells. The mechanism of uptake was elucidated, demonstrating that LNCs are incorporated mainly by caveolae-mediated endocytosis.

**Conclusions:**

We demonstrated that LNCs can be directed towards immunosuppressive cells by simply modulating their size and charge thus providing a novel approach to exploit nanosystems for anticancer treatment in the frame of immunotherapy.
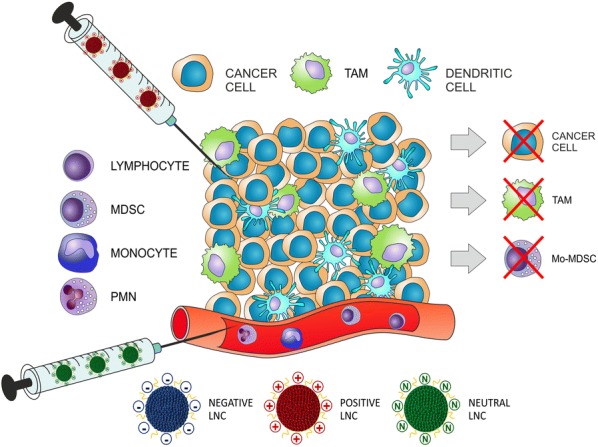

## Background

The recent implementation of nanosystems with different chemical and physical features and loaded with a variety of compounds offers promising opportunities to target selected cell populations in cancer [1]. The improved anti-tumor effects of nanomedicine has been widely ascribed to the direct cytotoxicity of chemotherapeutics on cancer cells, due to elevated drug concentrations in tumor tissue via passive- and/or tumor-targeting and favorable pharmacokinetics. In addition, the manipulation of the immune system significantly affects the efficacy of cancer therapies. The contribution of nanomedicine to either direct stimulation of the immune system by immunogenic cell death or reduction in immunosuppressive populations has the potential to increase antitumor immune response by regulating specific pathways within immune cell populations acting on their composition, geometry, or surface properties [2–4].

So far, the effect of size and surface charge has been explored for nanostructures targeting tumoral tissues and tumor microenvironment [5]. In regard to the impact of particle size on solid tumors, only nanoparticles smaller than 100 nm accumulated efficiently in poorly permeable tumors [6], while surface potential influences nanoparticle recognition by blood circulating and tissue phagocytes [7]. However, only few studies have explored the effect of these parameters on immunosuppressive myeloid cells.

Myeloid-derived suppressor cells (MDSCs) and tumor-associated macrophages (TAMs) constitute two of the main players involved in the induction of immune tolerance in cancer patients. MDSCs are a heterogeneous population of myeloid cells able to inhibit innate and adaptive immunity in cancer patients and mouse models [8]. Several populations of human MDSCs have been described, which, based on their phenotypic and morphological features, can be divided into three main subsets: monocytic MDSCs (M-MDSCs), polymorphonuclear MDSCs (PMN-MDSCs) and early-stage MDSCs (eMDSCs) [8]. TAMs are particularly abundant in the tumor mass of different tumors and can rapidly change their phenotype and function in response to local environmental stimuli, acquiring immunosuppressive and pro-tumoral properties [9] and hence they have been associated to poor clinical outcome [10]. Selective targeting of myeloid cells has been advanced as therapeutic strategy to relieve immunosuppression in patients and increase the response to conventional and immunotherapy treatments [11]. In this context, identification of a nanosystem selectively targeting tumor-promoting myeloid cells could represent a new tool to block their activity with the potential to be used in combination therapy with immune stimulating agents.

We previously demonstrated in a glioma rat model that lipid nanocapsules (LNCs) loaded with paclitaxel were able to inhibit multidrug resistance in glioma cells and to reduce tumor progression [12, 13]. Moreover, we also showed that an LNC formulation is endowed with a preferential targeting of myeloid cells. We found that LNCs loaded with a lauroyl-modified form of gemcitabine (GemC12) were able to target M-MDSCs, attenuate tumor-associated immunosuppression, and increase the efficacy of adoptive T cell therapy in lymphoma and melanoma-bearing mice. Moreover, the treatment of monocytes from melanoma patients with GemC12-loaded LNCs reduced their immunosuppressive properties in vitro [14].

Starting from these results, in this work we studied the impact of the physico-chemical properties, namely size and surface potential, of LNC formulations, already tested in mouse models [13–15], to increase their targeting abilities towards MDSCs and TAMs freshly isolated from glioblastoma (grade IV glioma, GBM) patients in which several immunosuppressive mechanisms have been documented. For example, the expansion of MDSC subsets has been reported in the peripheral blood of these patients as compared to healthy donors (HDs) [16], while at the tumor site, an abundant infiltrate of myeloid origin has been observed, mainly characterized by macrophages [17, 18], which were shown to possess immunosuppressive activity toward T cells [18–21]. TAMs comprise both resident microglia (MG) and macrophages of blood origin (bone marrow-derived macrophages-BMDM), and we recently demonstrated that in the center of GBM tumor mass BMDMs are abundant and endowed with a strong immune suppressive activity [18]. We therefore selected GBM as a model to test the targeting of optimized nanosystems towards immunosuppressive cells, with the future goal of loading these nanoparticles with selected drugs able to deplete or inhibit the activity of MDSCs and TAMs.

## Results

### The LNC internalization by blood leukocyte subsets depends on particle size

Given the importance of T cell activation and the negative role of myeloid cells in anti-tumor immune response, we rationally modified LNC formulations to target myeloid cells, while reducing the uptake by T cells. LNCs, prepared as previously described, were composed of FDA-approved excipients showing a good safety record and a tunable size (between 20 and 100 nm) [22, 23]. In this study, we tested LNCs with different physico-chemical properties, i.e. variable size and surface charge, to modulate cell uptake [4, 24, 25].

**Fig. 1:**
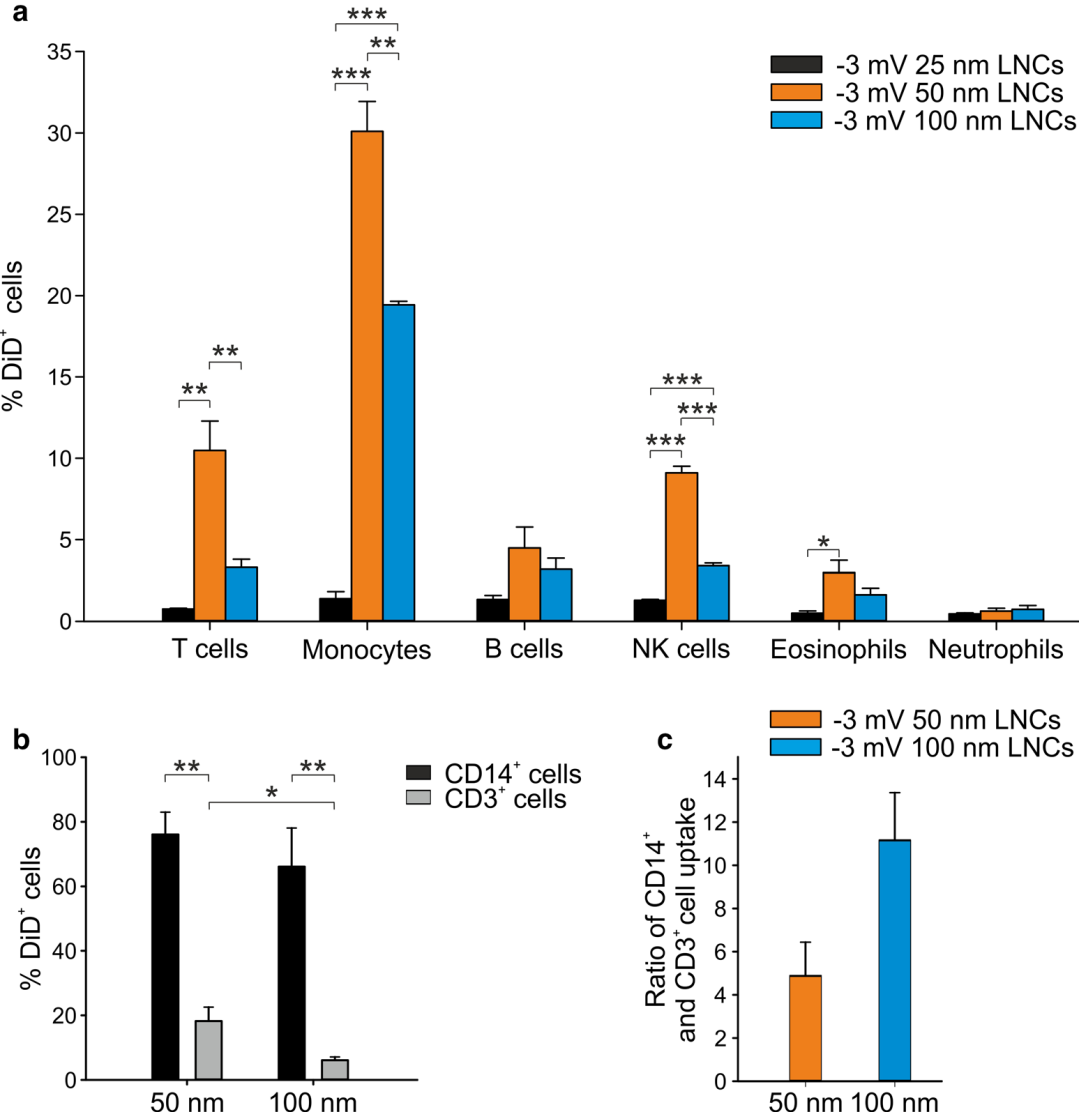
Incorporation of LNCs of different size by peripheral blood leukocytes (PBLs). **a** PBLs from 3 HDs were treated for 90′ with neutral DiD-LNCs of different size (25 nm in black, 50 nm in orange and 100 nm in blue), with DiD at 50 ng/ml and then stained with mAbs (anti-CD3, anti-CD14, anti-CD19, anti-CD56, anti-CD11b, anti-CD16) for flow cytometry analysis. Blank-LNCs were used as negative control. **b** PBLs from 3 HDs were treated for 3 h with 50 nm and 100 nm DiD-LNCs at a DiD concentration of 50 ng/ml and stained with anti-CD14 and anti-CD3 mAbs to identify monocyte (black) and T lymphocyte (grey) uptake. **c** The histogram in panel B shows the ratio between the percentage of DiD^+^ cells among CD14^+^ and CD3^+^ populations. Mean and standard error (SE) of 3 independent experiments are reported. Student t-test was performed, *P ≤ 0.05; **P ≤ 0.01; ***P ≤ 0.001

First, we tested the uptake of LNCs with 25 nm, 50 nm, and 100 nm size, neutrally charged (Table 1), and loaded with the fluorescent dye DiD (DiD-LNC). Incorporation by leukocyte subsets present in the peripheral blood of HDs was evaluated by multicolor flow cytometry, with mAbs directed against markers present on the cell surface of different leukocyte cell subsets and by assessing the signal emitted by DiD in T cells (CD3^+^), monocytes (CD14^+^), B cells (CD19^+^), NK cells (CD56^+^), eosinophils (CD11b^+^ CD16^−^), and polymorphonuclear cells (PMN, CD11b^+^ CD16^+^). Blank-LNCs were used as control. As shown in Fig. 1a, the incorporation of 25 nm LNCs was very low in all the considered leukocyte populations, while 50 nm LNCs showed the highest uptake in the analyzed subsets and in particular in monocytes (30.1 ± 3.2%). The 100 nm LNCs did not reach the same uptake of 50 nm LNCs on monocytes (19.4 ± 0.4%) but allowed the reduction in internalization by T lymphocytes (10.5 ± 3.1% with 50 nm LNCs *vs* 3.3 ± 0.9% with 100 nm LNCs). We excluded from the analysis the 25 nm LNCs and further investigated the internalization properties of neutral 50 nm and 100 nm LNCs, focusing on monocytes and T cells, and increasing the incubation time from 90′ to 3 h in order to reach the highest LNC internalization (Fig. 1b). Under these experimental conditions, both LNC formulations reached comparable high levels of internalization in monocytes, but the incorporation by T cells was significantly lower when 100 nm LNCs were used (Fig. 1b). By calculating the ratio between the signal of DiD in monocytes and T cells, we observed that 100 nm LNCs allowed increasing specificity of LNC targeting towards monocytes (mean ratio of 4.9 ± 2.7 for 50 nm LNCs vs 11.2 ± 3.8 for 100 nm LNCs) (Fig. 1c). Therefore, neutral 100 nm LNC formulation was chosen for further experiments.

**Table 1:** Size (nm), Polydispersion Index (PI) and Zeta Potential (mV) of LNCs (n > 3)

Formulation	Size (nm)	PI	Zeta Potential (mV)
25 nm LNCs neutral	25 ± 1	< 0.1	0 ± 1
50 nm LNCs neutral	53 ± 4	< 0.1	− 4 ± 1
100 nm LNCs neutral	101 ± 3	< 0.1	− 3 ± 0.5
100 nm LNCs negative	102 ± 3	< 0.1	− 20 ± 3
	99 ± 3	< 0.1	+ 6 ± 2
	102 ± 1	< 0.1	+ 16 ± 3
100 nm LNCs positive	92 ± 7	< 0.1	+ 25 ± 2
	95 ± 8	< 0.1	+ 31 ± 3

### Effect of 100 nm LNC surface charge on the internalization ability of peripheral blood leukocytes (PBLs)

We next set out to assess the surface charge of 100 nm LNCs to increase the specific uptake by monocytes compared to all the other main leukocyte populations. To this aim, we compared 100 nm neutral LNCs (− 3 mV) to LNCs with a slightly positive surface charge. The loading of cationic surfactant DDAB in nanosystems did not alter the size of the systems, while it affected the surface properties of the LNCs. The physico-chemical characteristics are summarized in Table 2.

**Table 2:** LNCs 25, 50 & 100 nm formulations

Excipient (mg)	LNC size (nm)		
	25	50	100
Labrafac^®^	600	1116.8	1800
Kolliphor^®^ HS15	1800	916.8	950
Span 80	300	450	300
MilliQ water	1300	1516.8	950
NaCl	54	54	54
Quenching water	2000	2000	2000

**Fig. 2:**
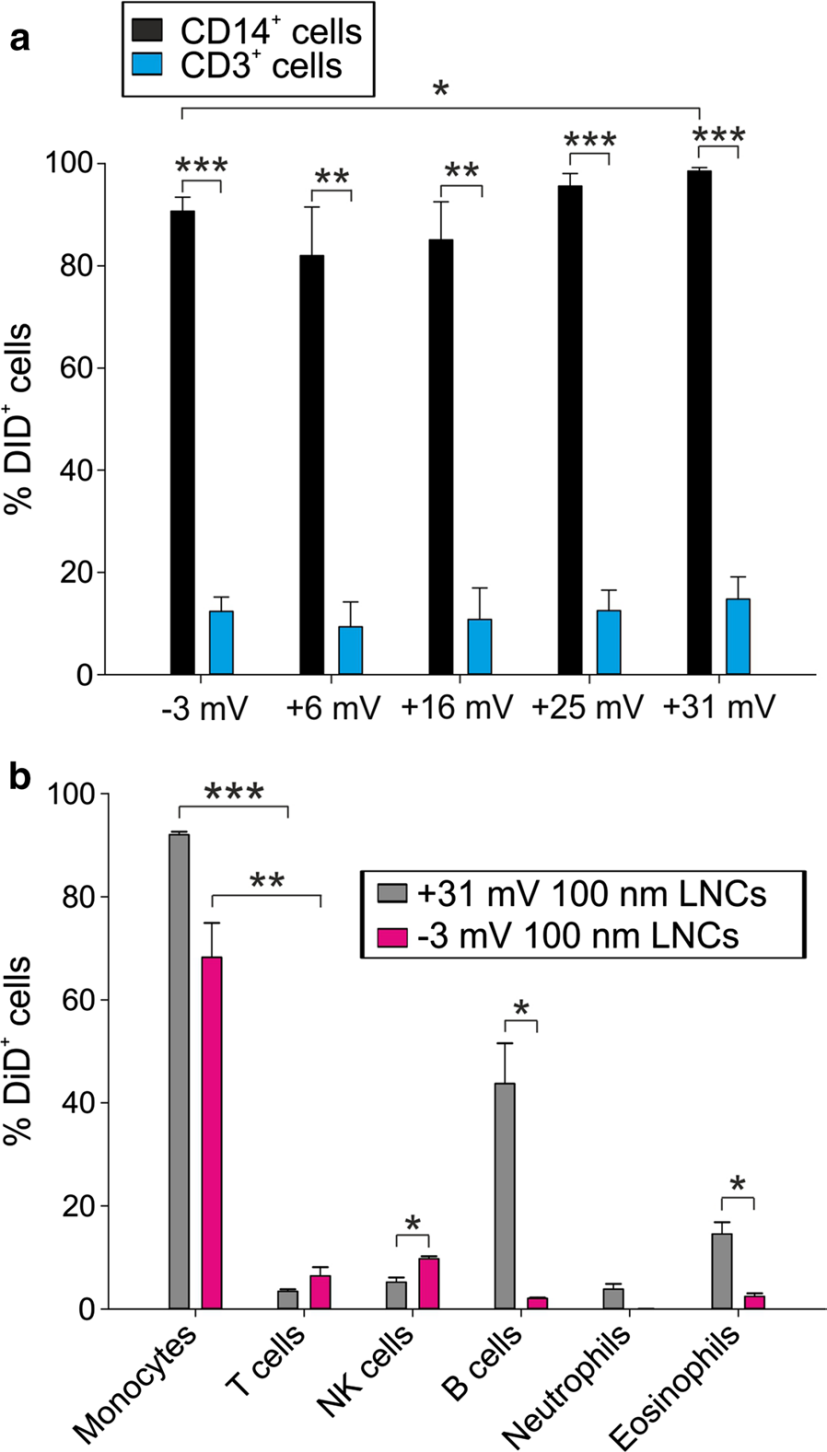
Incorporation of differently charged LNCs by leukocyte populations. **a** Mean and SE of three independent experiments on PBLs obtained from peripheral blood of HDs. 100 nm DiD-LNCs with different surface charge (from neutral to positive) at 50 ng/ml DiD concentration were incubated for 3 h with PBLs; DiD-LNC uptake was assessed on CD14^+^ (in black) and CD3^+^ cells (in blue). Blank-LNC samples were used as negative control. **b** 100 nm positively charged LNCs (+31 mV, grey) and neutral LNCs (− 3 mV, pink) were incubated with PBLs for 3 h at 50 ng/ml DiD concentration. Did^+^ cells by flow cytometry analysis. Mean and SE of 3 independent experiments are reported. Student t-test was performed, *P ≤ 0.05; **P ≤ 0.01; ***P ≤ 0.001

After 3 h of incubation of PBLs with DiD-loaded LNCs (Fig. 2a), the internalization by T cells was very low and comparable in all the tested LNC formulations, while in monocytes the incorporation was always significantly higher than that of T cells, and had a trend toward an increase as the positive charge augmented (98.6 ± 1.2% of DiD^+^ monocytes using +31 mV LNCs vs 90.7 ± 4.7% with neutral LNCs; 82.0 ± 16.5% with + 6 mV LNCs; 85.1 ± 12.9% with +16 mV LNCs; 95.6 ± 4.3% with +25 mV LNCs).

We therefore tested the internalization of 100 nm positive LNCs (+ 31 mV) by all leukocyte subsets present in the peripheral blood of HDs and compared the results to neutral LNC formulation (Fig. 2b). Monocytes showed the highest incorporation of positive (+31 mV) LNCs (92.0 ± 1.4%), but a high uptake was also noticed for B lymphocytes (43.7 ± 19.2%), an effect that was not observed using neutral LNCs (2.1 ± 0.3%). This could lead to a deleterious consequence on the patient’s immune response if LNCs loaded with a cytotoxic drug were used. We thus selected the 100 nm neutral surface charge formulation to target monocytes in the peripheral blood and avoid incorporation by B cells.

### Targeting circulating immunosuppressive cells in GBM patients by 100 nm neutral LNCs

**Fig. 3:**
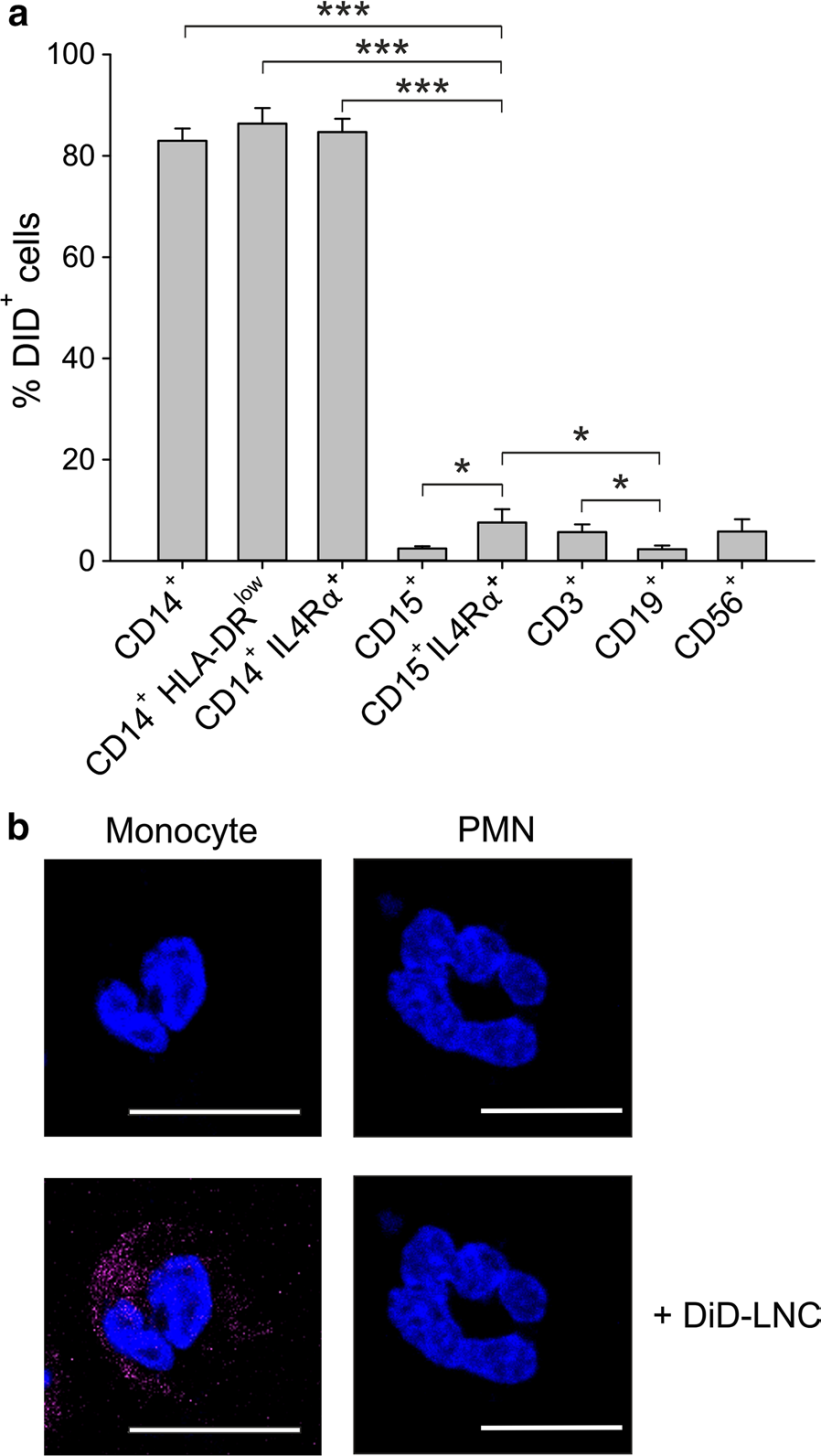
Uptake of neutral LNCs by leukocyte subsets in the peripheral blood of GBM patients. **a** Mean and SE of PBLs from 7 GBM patients incubated with 100 nm neutral LNCs for 3 h at a DiD concentration of 50 ng/ml. DiD-LNC uptake was evaluated by flow cytometry. Mann–Whitney test was performed, *P ≤ 0.05; **P ≤ 0.01; ***P ≤ 0.001. **b** PBMCs and PMNs isolated from GBM patients were incubated with 100 nm neutral LNCs for 3 h at a DiD concentration of 50 ng/ml. Then cells were washed and plated, nuclei were counterstained with DAPI, and the slides were analyzed by confocal microscopy. Representative fluorescence images (monocyte and PMN) are shown at a magnification of 150X. Cell size is reported by scale bar (10 µm)

Data from literature [16] and our own results (Pinton et al., unpublished) indicate that GBM patients have a significant expansion in circulating MDSC populations. We thus tested the uptake of the 100 nm neutral LNC formulation on leukocyte subsets present in the peripheral blood of these patients, extending the analysis to three MDSC subsets: two monocytic subsets (identified as CD14^+^ IL4Rα^+^ and CD14^+^ HLA-DR^low^ cells) and one PMN type (CD15^+^ IL4Rα^+^) [8]. Following PBL incubation with DiD-loaded LNCs, maximum LNC internalization was observed by total monocytes (CD14^+^ cells: 83.0 ± 6.4%) and by monocyte subsets CD14^+^ HLA-DR^low^ (86.4 ± 8.2%) and CD14^+^ IL4Rα^+^ (84.7 ± 6.9%) cells, corresponding to monocytic MDSCs (Fig. 3a), thus highlighting that this nanocarrier system could efficiently target immunosuppressive myeloid cells in GBM patients, while sparing lymphocyte subsets that, instead, showed very low uptake. A lower level of incorporation was observed in PMN-MDSCs, defined as CD15^+^ IL4Rα^+^ cells, as compared to the two monocytic subsets; it should be noted that this immunosuppressive granulocytic population shows a significantly higher LNC uptake as compared to total PMNs (defined as CD15^+^ cells) (7.6 ± 7% DiD^+^ cells in CD15^+^ IL4Rα^+^ vs 2.4 ± 1.2% DiD^+^ cells in PMNs), thus reinforcing the efficacy of LNCs in targeting immunosuppressive cells. To confirm intracellular localization of LNCs, we performed confocal analysis in isolated peripheral blood mononuclear cells (PBMCs) and PMN fractions, and observed that DiD-loaded lipid nanoparticles are internalized by monocytes showing cytoplasmic localization, while no uptake by granulocytes was observed (Fig. 3b).

### Mechanism of LNC internalization by circulating monocytes

**Fig. 4:**
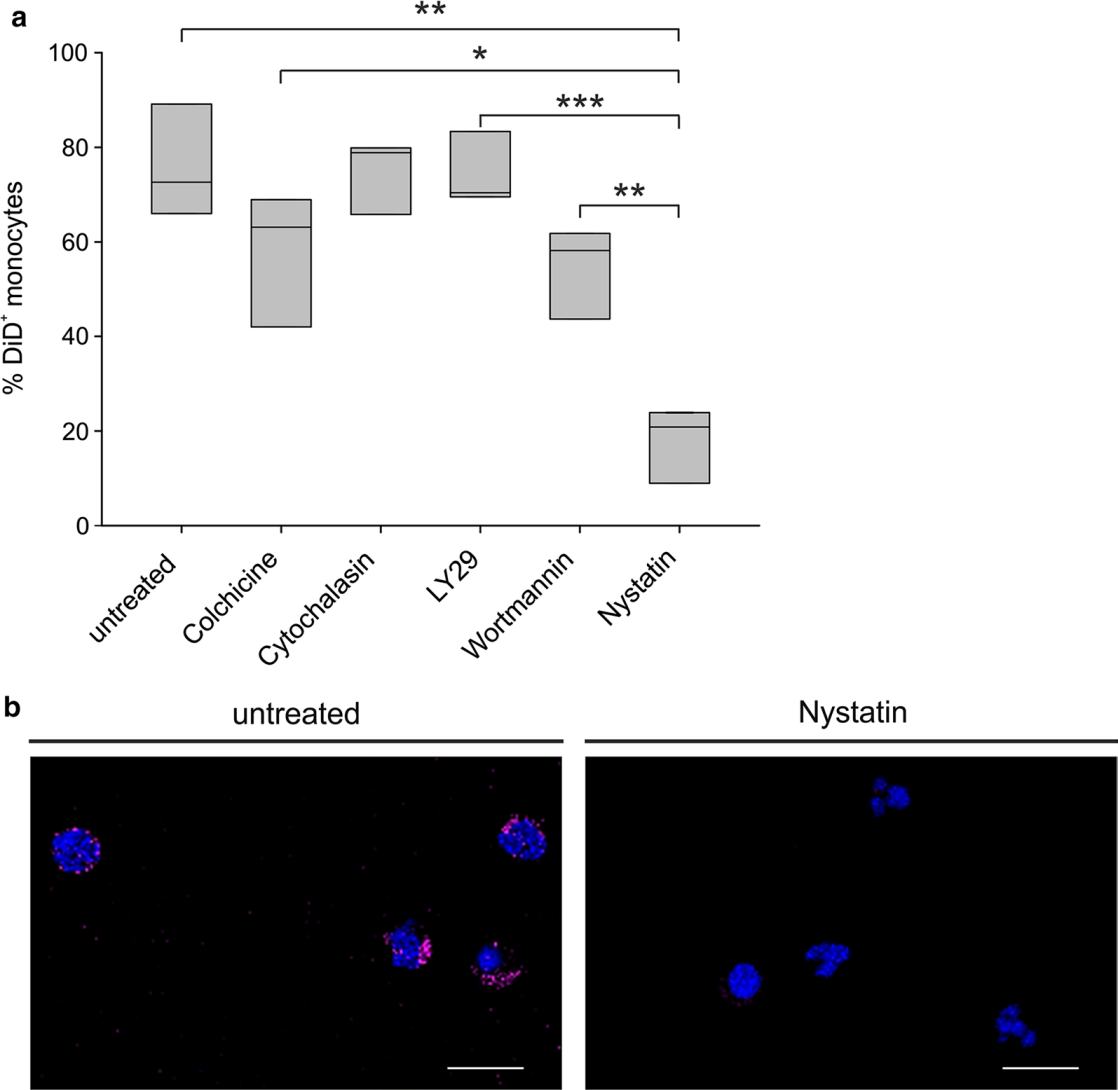
Effect of different inhibitors on LNC uptake by circulating monocytes. **a** PBLs from 3 GBM patients were incubated for 2 h with 100 µg/ml colchicine or 10 µg/ml cytochalasin B, 45′ with 50 µM LY294002, 30′ with 100 nM Wortmannin, 15′ with 100 U/ml Nystatin. DiD-loaded LNCs were added to PBLs at 50 ng/ml DiD for 3 h and then stained for cytometry analysis. Box plots show the range of DiD^+^ cells in the subsets analyzed in 3 experiments. Student t-test was performed, *P ≤ 0.05; **P ≤ 0.01; ***P ≤ 0.001. **b** Representative images of confocal analysis of PBMCs from 3 GBM patients incubated with Nystatin and DiD-loaded LNCs at 50 ng/ml DiD for 3 h. Cells were stained with DAPI, and slides were analyzed at a 63× magnification. Cell size is reported by scale bar (10 µm)

To gain evidence about the mechanisms involved in 100 nm neutral LNC internalization by monocytes, PBLs from GBM patients were treated with inhibitors of different uptake mechanisms and their effect was verified on particle internalization. Colchicine was used to inhibit pinocytosis [26], cytochalasin B as inhibitor of phagocytosis [26], LY294002 and Wortmannin as inhibitors of fluid phase pinocytosis and FcR-mediated phagocytosis [27, 28], and nystatin as inhibitor of caveolae-mediated endocytosis [29]. Results indicate that nystatin is the most effective inhibitor, causing a significant reduction in the uptake of monocytes (Fig. 4a). Another inhibitor that showed a trend toward a reduced LNC internalization by monocytes is colchicine, although its effect did not reach the same level observed with nystatin, lacking statistical significance. Altogether, these results indicate that 100 nm neutral LNCs are mainly internalized by monocytes from GBM patients through caveolae-mediated endocytosis. We also visualized the effect of nystatin on LNC uptake by PBMCs from three glioma patients, by means of confocal microscopy. Monocytes were enriched by adhesion onto microscope slides and further selected by nuclear morphology; following nystatin treatment, we observed a remarkable reduction in the internalization of DiD-loaded LNCs (Fig. 4b), thus confirming the results obtained by flow cytometry.

### Evaluation of LNC uptake by the cells present in GBM microenvironment

**Fig. 5:**
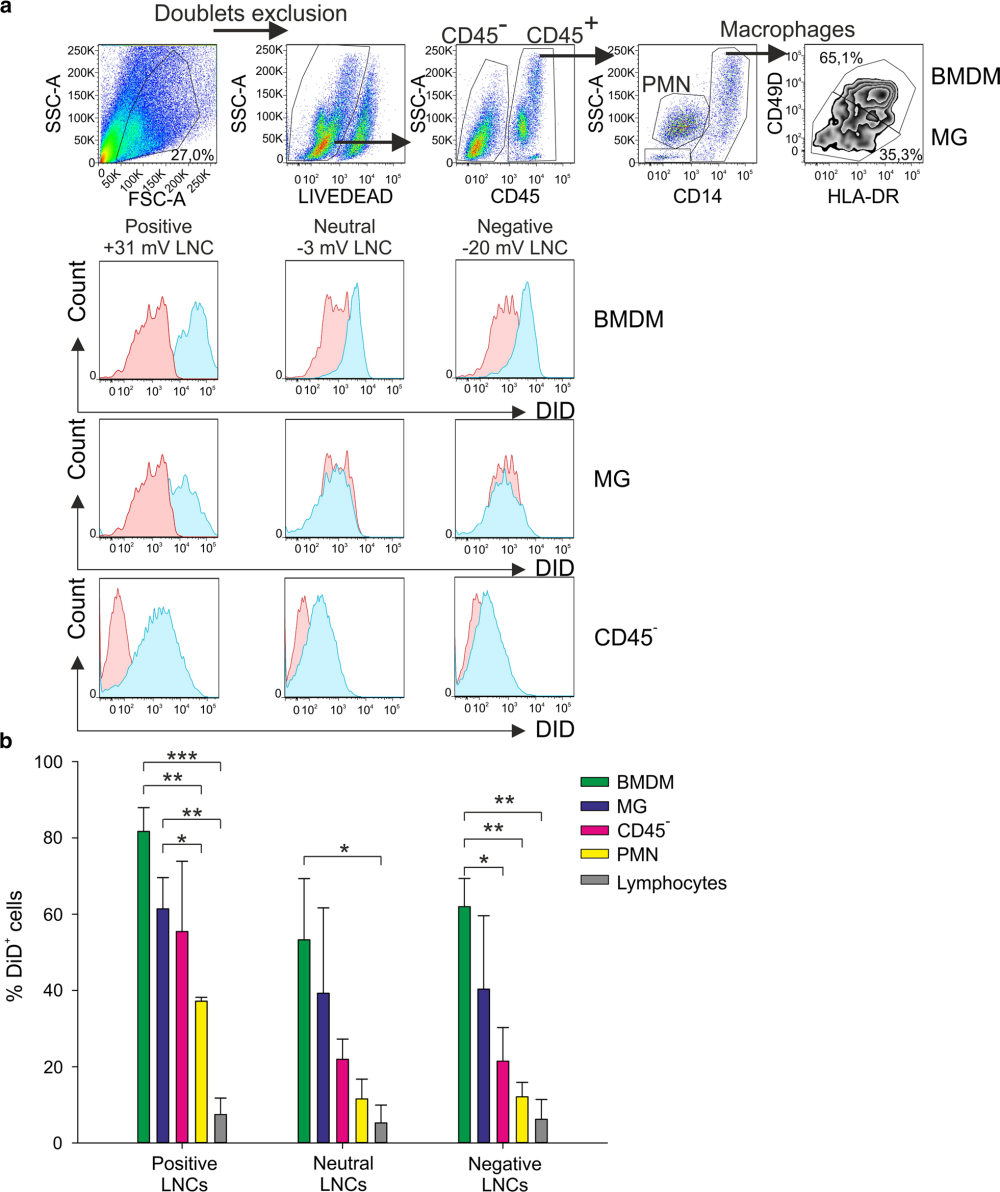
Uptake of differently charged LNCs by the cells in GBM microenvironment. **a** Representative example of flow cytometry analysis on cell suspension from GBM tissue incubated overnight with 100 nm negative, neutral and positive DiD-LNC formulations at 50 ng/ml DiD concentration. The gating strategy is reported in the upper panels. DiD^+^ cells (blue histograms) were assessed among BMDM, MG, CD45^−^ cells and compared to the signal of Blank-LNCs (red histogram) among the same populations. **b** The histograms show mean ± SE of 3 independent experiments, performed as described in (A). The percentage of DiD^+^ cells was calculated in BMDM (green), MG (blue), CD45^−^ cells (pink), PMN (yellow), and lymphocytes (grey), setting the gate on Blank-LNC control. PMNs were gated as CD14^−^ SSC^high^ cells and lymphocytes as CD14^−^ SSC^low^ cells. Student t-test was performed, *P ≤ 0.05; **P ≤ 0.01; ***P ≤ 0.001

Since 100 nm neutral LNCs efficiently target myeloid cells in the blood of GBM patients, we evaluated whether the same nanosystem could be incorporated by different cell subsets present in the tumor microenvironment. In fact, our recent results highlight the presence of an abundant leukocyte infiltrate of myeloid origin, mainly constituted by macrophages, as a main characteristic in GBM. We demonstrated that only macrophages of bone marrow origin, and not the resident microglial cells (MG), were endowed with a strong immunosuppressive activity [18]. In fact, during tumor growth, macrophages originating from bone-marrow (BMDM) are recruited to the tumor, and can be distinguished by tissue resident MG through a marker combination in multicolor flow cytometry (Fig. 5a upper panels) [17, 18]. We therefore tested the incorporation of different LNC formulations with a size of 100 nm, carrying overall positive, neutral or negative charge, in the cell suspension freshly obtained from tumor tissue and analyzed the incorporation by BMDM, MG, tumor cells (CD45^−^ cells), PMNs, and lymphocytes. The maximum LNC uptake was obtained for macrophages (BMDM and MG) and tumor cells when positively charged LNCs were used; the low level the incorporation by T cells was comparable across different formulations. Moreover, both BMDM and MG cells also reached an uptake significantly higher than PMNs and this LNC formulation also allowed an increased uptake by tumor cells, evaluated as CD45^−^ cells (Fig. 5a and b).

Positively charged LNCs show a very high uptake by B cells from the peripheral blood (Fig. 2b), but B lymphocytes are not present in GBM tumors (data not shown); therefore, these data indicate that 100 nm positively charged LNCs could be used as a drug-loaded nanosystem to target the main immune suppressive cell subset in these tumors. Moreover, tumor cells also show a significant uptake, thus reinforcing the possibility of using this nanosystem to target both tumor cells and tumor-promoting cells.

### Mechanism of LNC internalization by the GBM microenvironment

**Fig. 6:**
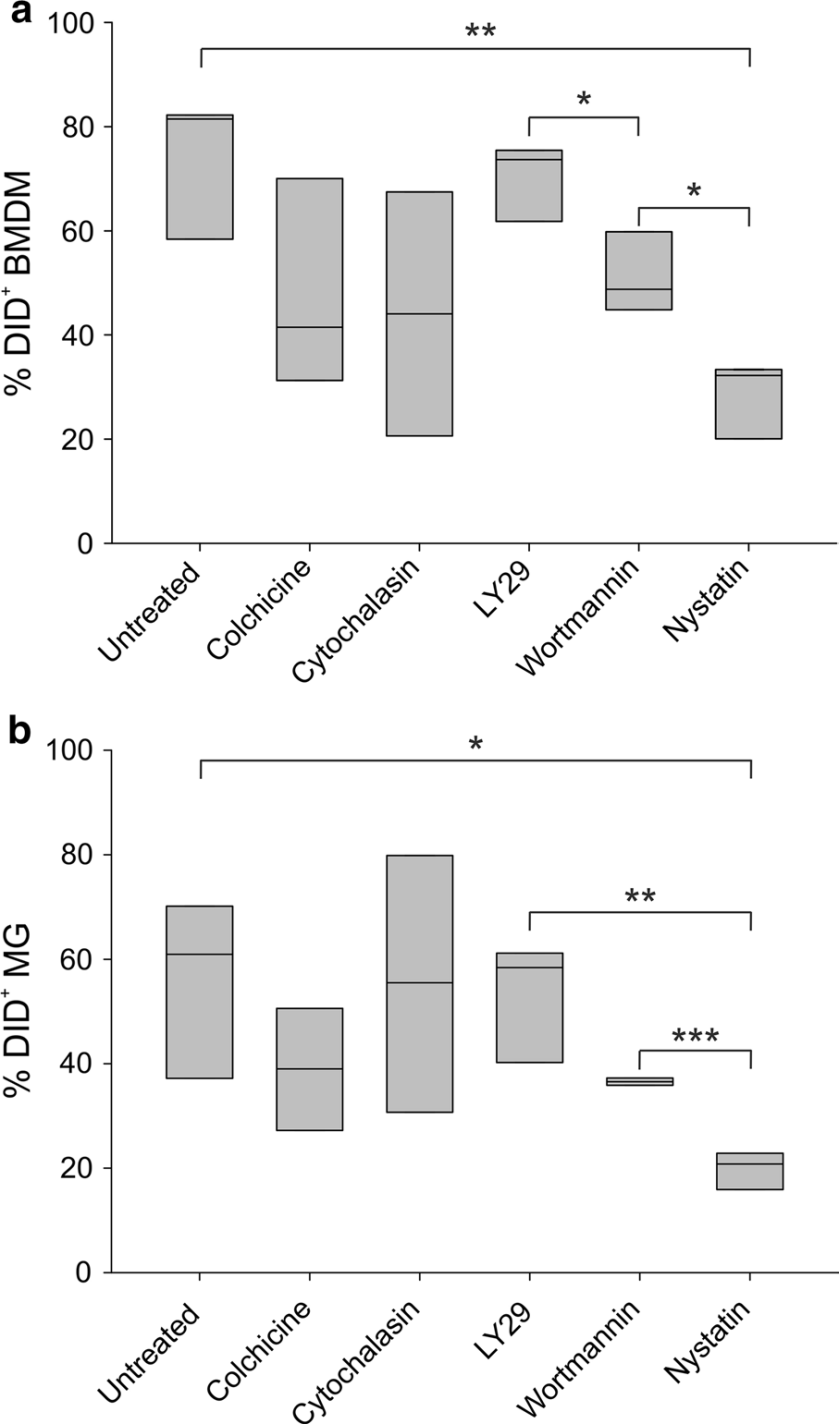
Mechanism of LNC internalization by BMDM and MG. Box plots show the range of DiD^+^ cells among BMDM **a** and MG **b** in 3 independent experiments. Cells from GBM tissue were incubated with 100 µg/ml colchicine, 10 µg/ml cytochalasin B, 50 µM LY294002, 100 nM Wortmannin, 100 U/ml Nystatin, DiD-loaded LNCs were added at 50 ng/ml DiD over-night and LNC internalization was analyzed by flow cytometry. Student t-test was performed, *P ≤ 0.05; **P ≤ 0.01; ***P ≤ 0.001

Given the high ability of tumor macrophages to internalize positively charged LNCs, we analyzed the mechanism by which these nanoparticles were incorporated by the cells of the tumor microenvironment. We thus treated the cell suspension obtained after enzymatic digestion of GBM tumor tissue with uptake inhibitors and added DiD-loaded LNCs to cell suspensions for overnight incubation. As shown in Fig. 6, internalization by both types of macrophages (BMDM and MG) was significantly reduced by the addition of nystatin, in line with the internalization observed by blood monocytes. A reduction in LNC uptake was also observed using colchicine in both BMDM and MG and cytochalasin B in BMDM, but without statistical significance.

## Discussion

Functionalization of nanosystems offers the opportunity to maximize the protection of associated drug and the targeting properties of the carrier towards the desired cell populations. In this study, we optimized LNCs to target immunosuppressive populations in GBM patients, with the aim of disclosing innovative applications in the field of immunotherapy. Indeed, as recently shown in two pioneer studies of personalized vaccination in GBM patients, the immune system can be activated toward tumor antigens expressed by the tumor but the induction of an immune response does not directly translate into a clinical benefit, likely because other critical aspects of the complex interaction between tumor and immune systems are not yet defined [30, 31]. From several studies, it appears that one of the main obstacles toward a successful anti-tumor immune response is the suppression exerted by myeloid cells on cells of the adaptive immune system. Therefore, we tested known LNC formulations, with slight modifications in size and charge, capable of targeting immune suppressive subsets in cancer patients, but we also evaluated the interactions of LNCs with many subsets of the immune system, including those potentially able to mediate cancer regression, like the T lymphocytes. To the best of our knowledge, this is the first study to provide a broad picture on the impact of different LNC formulations on human immune cell subsets freshly isolated from GBM patients. We thus extend our previous results focused on immune suppressive myeloid cells in the blood of melanoma patients to different populations of circulating and tumor-associated myeloid cells in GBM patients. The LNC formulations used in the present work were already tested in mouse models and proved to be non-toxic, using different administration routes, and effective in reaching tumor site and reducing tumor growth [13–15].

Until now, only a few studies addressed LNC targeting towards MDSCs as a way to either deplete them or to induce their maturation. Aptamer (T1) conjugated to liposomal doxorubicin [32] or lipid-coated biodegradable hollow mesoporous silica nanoparticle co-encapsulated with all-trans retinoic acid (ATRA), doxorubicin and IL-2 [33] showed a high affinity for both tumor cells and PMN-MDSCs [32] or to induce a reduction in the number of MDSCs in the tumor microenvironment of mouse models [33]. However, these systems showed no affinity for M-MDSCs or macrophages therefore reducing their applicability only to tumors in which PMN MDSCs are mainly involved [32]. Besides, they affected other cell subsets present in the tumor microenvironment, and thus cannot be considered a nanosystem with a specific targeting towards MDSCs.

Moreover, Zinc-doped iron oxide nanoparticles modified with polyethylenimine molecules and dimercaptosuccinic acid in combination with radiotherapy prolonged survival of CT-2A mouse glioma model and were mainly incorporated by TAM/MDSCs, although the definition of these cell populations was based only on CD45 and CD11b markers without further characterization [34]. The activity of MDSCs was shown to be modulated also using polyarginine nanocapsules carrying the chemokine CCL2 and an RNAi sequence targeting C/EBPβ, a transcriptional factor fundamental for MDSC differentiation and functions [35], thus showing that MDSCs represent an interesting target in a nanomedicine approach.

The idea of using tailored LNCs, encapsulated with selected drugs, to reduce blood monocytes stems from the findings that monocytes are actively recruited at tumor site and sustain the accumulation of immunosuppressive macrophages in the tumor microenvironment of GBM patients [18]. Given the high rate of relapse in GBM patients, targeting blood monocytes should be evaluated as an adjuvant therapy in such patients after surgical resection, to deplete them or to block their function. This strategy would allow the inhibition of the loop through which GBM tumor attracts immunosuppressive cells at tumor site and suppresses the anti-tumor immune response. The stability of neutral 60 nm LNCs loaded with 5-FU in human plasma was already tested and proved that the system is stable and protected the drug from rapid degradation, thus confirming the potential use of LNCs as a tool to target immunosuppressive cells in peripheral blood of patients [36].

The efficacy of LNCs in the treatment of glioma was demonstrated in GL261 glioma-bearing mice following stereotactic injection. Multifunctional lipid nanocapsules designed to combine the activity of the cytotoxic drug paclitaxel (PTX) with the immunostimulant CpG were intratumorally administered and were able to increase the survival of mice compared to control, i.e. the free Taxol^®^, or PTX-loaded LNCs. This effect was also confirmed by magnetic resonance imaging, which revealed the reduction of tumor growth in the treated animals [13]. Moreover, Vanpouille-Box et al. demonstrated that lipid nanocapsules loaded with rhenium-188 (LNC^188^ Re-SSS) implanted in the brain of a rat orthotopic glioma model triggered remarkable survival responses. A strong activation of myeloid cells assessed by immunohistochemistry was observed in this model together with the recruitment of natural killer and dendritic cells, thus suggesting an improved capacity to develop an antitumor immune response [37]. Another study, performed in a murine glioma model with a different type of nanosystem, i.e. cyclodextrin-based nanoparticle (CDP-NP), demonstrated a predominant uptake of CDP-NP by macrophages and microglia within and around the tumor site [38].

In the present study we optimized an LNC formulation to target immunosuppressive BMDMs in the GBM microenvironment, but documented that also tumor cells incorporate a lower but significant amount of such LNCs, thus demonstrating that this nanosystem might target both tumor cells and tumor-promoting cells. LNC incorporation was observed to a lower extent also in resident MG cells but, since it has been demonstrated that in GBM these cells display an activated phenotype that promotes tumor progression [39] and in the center of tumor lesion they exert a moderate immunosuppressive activity, their targeting by drug-loaded LNC could also be beneficial [18]. Further studies will be required to investigate the effect of these nanoparticles on other immune cell subsets, such as dendritic cells, that have a key role in the modulation of the immune system and are expected to phagocyte LNCs, therefore becoming one of their targets [40]. These results open the road to new strategies of therapeutic interventions. For example, LNCs could be loaded with a drug inducing immunogenic cell death, offering the possibility of eliminating both suppressive macrophages and tumor cells, thus reversing a tolerogenic microenvironment, and providing the rationale for a new treatment of GBM. However, systemic administration would lead to a high uptake by liver, kidney, and spleen, and would not guarantee a sufficient uptake by the tumor [41, 42]. Thus, to avoid this problem, an intra-thecal administration, following tumor resection, could be exploited, in order to maximize local efficacy and reduce systemic side effects.

## Conclusions

Our study shows that modulation of size and charge of nanosystems impacts the uptake by human blood circulating leukocytes and tumor-infiltrating cells. We exploited such properties to optimize LNC targeting towards immunosuppressive populations, while maintaining a low level of internalization by T cells. We propose neutral 100 nm LNCs and positively charged 100 nm LNCs as the most effective targeting nanosystems respectively in the blood and at tumor site in glioblastoma patients. This study represents a proof of principle that different physico-chemical characteristics of a nanocarrier system can be exploited to target a specific cell subset, while sparing others of therapeutic importance. This approach could be extended to other cancers and set the ground as a new tailored anticancer treatment in the frame of immunotherapy.

## Methods

### Patient characteristics

Patients were recruited at the Department of Neurosurgery, Padova University Hospital, Italy. The ethical committee of the IOV-IRCCS and of Padova University Hospital approved all experiments and all patients gave their informed consent. We analyzed peripheral blood from 12 GBM patients and tumor tissue from 6 GBM patients. The studies were conducted in accordance with the Declaration of Helsinki. To optimize experimental conditions and to test the internalization properties of different LNC formulations, peripheral blood of 15 HDs was analyzed.

### Isolation of PBLs and PBMCs from peripheral blood of HDs and GBM patients

Peripheral blood was collected from HDs and GBM patients and subjected to lysis to remove red blood cells and viable PBLs were counted. PBMCs and PMNs were isolated as previously described [43]. Additional information are described in Additional file 1: Supplementary methods.

### GBM tissue processing to obtain a single-cell suspension

GBM tumors were processed immediately after resection, as described in Additional file 1: Supplementary methods, to obtain a single cell suspension without erythrocytes and debris.

### Preparation of lipid nanocapsules

LNC formulation was based on a phase inversion process already described [14, 22]. Different LNCs having a size of 25, 50, and 100 nm were prepared by varying component amounts according to Table 2.

### Physico-chemical characterization of LNCs

Size, estimated by the average hydrodynamic diameter, polydispersity index (PDI) and zeta (ζ)-potentials were determined by dynamic light scattering (DLS) using NanoZS^®^ (Malvern Instruments, Worcestershire, United Kingdom). Electrophoretic mobility was converted to ζ-potentials using Smoluchowski’s equation. Measurements were performed at a 173° angle after dispersion of 50 μL LNCs in 2.95 ml MilliQ water to ensure convenient scattered intensity on the detector. All measurements were performed in triplicate at 25 °C with comparable conductivity for ζ-potential determination.

### LNC incorporation by cell populations in peripheral blood and tumor tissue

PBLs, PBMCs, or tumor cell suspension obtained as previously described were incubated with LNCs at the final DiD concentration of 50 ng/ml for 90′, 3 h, or overnight at 37 °C. As control, Blank-LNC formulations were used. 10 µg/ml Cytochalasin B (Sigma-Aldrich), 100 µg/ml Colchicine (Sigma-Aldrich), 50 µM LY294002 hydrochloride (Sigma-Aldrich), 100 nM Wortmannin (Sigma-Aldrich), and 100 U/ml Nystatin were used to test the mechanism of internalization. At the end of the incubation, cells were stained for flow cytometry analysis. Further details are reported in Additional file 1: Supplementary methods.

### Multiparametric flow cytometry

Leukocytes were stained with antibody cocktails in order to define different cell populations as specified in Additional file 1: Supplementary materials and methods. Data acquisition was performed using LSRII flow cytometer (BD Biosciences) and results were analyzed by FlowJo software (Three Star Inc). Further details in Additional file 1: Supplementary methods.

### Confocal microscopy

PBMCs and PMNs were incubated with LNCs and nystatin inhibitor and then prepared for confocal analysis as specified in Additional file 1: Supplementary methods. Samples were analyzed under a laser scanning confocal microscope (Leica TCS SP5, Wetzlar, Germany) equipped with 4 lasers (405 nm/Argon-458,476,488,494,514 nm-/561 nm/633 nm), and results were analyzed by Las X (Leica MICROSYSTEMS).

### Statistical analysis

The Mann–Whitney and the Student t-test were used as appropriate and performed by Sigmaplot software (Systat Software Inc., CA, USA).

## Supplementary information

**Additional file 1.** Supplementary materials and methods. Isolation of cell populations from peripheral blood and tissue of GBM patients, preparation of lipid nanocapsules, LNC incorporation studies, multiparametric flow cytometry, confocal microscopy, statistical analysis.

## Data Availability

Not applicable.

## Abbreviations

ATRA: All-trans retinoic acid
BM: Bone-marrow
BMDM: Bone marrow-derived macrophages
eMDSC: Early-stage MDSC
GBM: Glioblastoma multiforme
GemC12: Lauroyl-modified form of gemcitabine
HD: Healthy donor
LNC: Lipid nanocapsule
MG: Microglia
MDSC: Myeloid derived suppressor cell
M-MDSC: Monocytic MDSC
PTX: Paclitaxel
PBMC: Peripheral blood mononuclear cell
PMN: Polymorphonuclear cell
PMN-MDSC: Polymorphonuclear MDSC
TAM: Tumor-associated macrophage
