# Supplementary material for: Targeting of immunosuppressive myeloid cells from glioblastoma patients by modulation of size and surface charge of lipid nanocapsules
Source: J Nanobiotechnology. 2020 Feb 17;18:31. doi: 10.1186/s12951-020-00589-3 (PMC7026969; doi:10.1186/s12951-020-00589-3)
Supplement: Supplementary file 1 — Additional file 1. Supplementary materials and methods. Isolation of cell populations from peripheral blood and tissue of GBM patients, preparation of lipid nanocapsules, LNC incorporation studies, multiparametric flow cytometry, confocal microscopy, statistical analysis. [file 12951_2020_589_MOESM1_ESM.docx]

**Additional files:**

**Additional file 1:**

**Supplementary Materials and methods.** Isolation of cell populations from peripheral blood and tissue of GBM patients, preparation of lipid nanocapsules, LNC incorporation studies, multiparametric flow cytometry, confocal microscopy, statistical analysis.

**Supplementary Materials and Methods**

**Isolation of PBLs and PBMCs from peripheral blood of HDs and GBM patients**

Peripheral blood was collected from HDs and GBM patients and subjected to lysis to remove red blood cells, with a hypotonic solution of ammonium chloride potassium composed of NH_4_Cl 1.54 M (Sigma-Aldrich), KHCO_3_ 0.01 Mm (Analytical Carlo Erba) and EDTA 0.5 M (Sigma-Aldrich). 2.5 ml of peripheral blood were lysed by adding 25 ml of lysing solution for 5’ at room temperature (RT). At the end of the incubation, lysing solution was diluted adding 10 ml of complete RPMI medium composed of RPMI (Roswell Park Memorial Institute Medium, Euroclone) culture medium supplemented with 10% of fetal bovine serum (FBS, Gibco, Thermo Fisher), 10 mM of Hepes (Lonza), 100 U/ml of Penicillin/Streptavidin (Lonza), 0.28 mM of Asparagine (Sigma-Aldrich), 1.5 mM of Glutamine (Sigma-Aldrich) and 0.67 mM of Arginine (Sigma-Aldrich). After centrifugation, cell pellet was resuspended in complete RPMI medium and viable PBLs were counted by trypan blue exclusion.

Peripheral blood mononuclear cells (PBMCs) were isolated from peripheral blood by density gradient centrifugation on Ficoll-Paque PLUS (GE Healthcare-Amersham, NJ, USA), as previously described [43], followed by dextran sedimentation to obtain PMNs, as previously described [43]. PBMCs and PMNs were resuspended in complete RPMI medium and counted by trypan blue exclusion.

**GBM tissue processing to obtain a single-cell suspension**

GBM tumors were processed immediately after resection. They were extensively washed with 0.9%

sodium chloride to remove peripheral blood, and then subjected to enzymatic digestion using human Tumor Dissociation Kit (Miltenyi Biotec) and gentleMACS^TM^ Octo Dissociator with heaters (Miltenyi Biotec), following manufacturer’s instructions for soft tumor digestion. If residual

erythrocytes were present, cell pellet was lysed by adding 10 ml of lysing solution for 3’ at RT. At the end of incubation, 10 ml of complete RPMI medium were added and, after centrifugation, cell pellet was resuspended in the same medium to count viable cells by trypan blue exclusion. If debris or myelin residues were present in cell suspension, they were removed in order to avoid interference

with LNC uptake, using debris removal solution (Milteny Biotec), following manufacturer’s instructions.

**Chemicals**

Labrafac® WL 1349 (Labrafac, caprylic-capric acid triglycerides) was provided by Gattefossé S.A.

(Saint-Priest, France). Kolliphor® HS15 (Kol, formerly Solutol® HS15; mixture of free polyethylene glycol 660 and polyethylene glycol 660 hydroxystearate) were supplied by BASF (Ludwigshafen, Germany). Deionized water was obtained from a Milli-Q plus system (Millipore, Paris, France). Span® 80 (Span 80), Tween® 80 (Tween 80), dodecanoic anhydride, sodium chloride, urea and acid lauric were purchased from Sigma (St Quentin-Fallavier, France). Ethanol, dichloromethane and methanol were purchased from Fischer Scientific (Loughborough, United Kingdom). Didodecyldimethylammonium bromide (DDAB) and Stearic acid (SA) were provided by Sigma (St Quentin-Fallavier, France). DID (1,1’-dioctadecyl-3,3,3’,3’- tetramethylindocarbocyanine 4-chlorobenzenesulfonate) was provided by Molecular Probes (R) (Eugene, OR).

**Preparation of lipid nanocapsules**

To prepare lipid nanocapsules, an initial emulsion constituted by the oil (Labrafac®), surfactant (Kolliphor®HS15 and Span®80), water, and NaCl was heated up to 75 °C under magnetic stirring and then cooled to 45 °C. This temperature cycle was repeated three times. During the last temperature decrease, at the phase inversion temperature (55 °C), an irreversible shock was induced

by dilution with pure water. Finally, a gentle magnetic stirring was maintained for 5’ [14]. The sample was stirred for 1h in a water bath at 60°C. Then, the same process described for the blank systems was carried out. Positive LNCs were prepared by adding 0.55, 1.1, 2.2 or 3.3% of DDAB (% m/m of Labrafac) to the oily mixture, negative LNCs were prepared by adding 3.3% of SA (% m/m of Labrafac) to the oily mixture. Fluorescent labelled LNCs were prepared using DiD as fluorescent probe. Briefly, 74 μl of DiD-acetone solution (5 mg/ml) were added in a vial to the oily mixture, then the LNCs were formulated as described above.

**LNC incorporation by cell populations in peripheral blood and tumor tissue**

PBLs, PBMCs, or tumor cell suspension obtained as previously described were resuspended in complete RPMI medium at a final concentration of 10^6^ cells/ml in polypropylene round-bottom tubes pre-coated with FBS in order to prevent cell adhesion. LNC formulations were diluted in the same medium in order to obtain the final DiD concentration of 50 ng/ml and incubated with the cells for 90’, 3 hours, or overnight at 37°C with 5% of CO_2_. As control, Blank-LNC formulations were used at the same LNC concentration of DiD-loaded LNCs (137 μg/ml). At the end of the incubation, cells were washed twice with culture medium to remove non-incorporated LNCs and then stained with fluorochrome-conjugated monoclonal antibodies (mAbs) to perform flow cytometry analysis.

To understand the mechanisms of LNC internalization, PBLs and tumor cell suspension were resuspended in complete RPMI medium at a cell concentration of 10^6^ cells/ml and incubated with 10 μg/ml Cytochalasin B (Sigma-Aldrich) for 2 h, 100 μg/ml Colchicine (Sigma-Aldrich) for 2h, 50

μM LY294002 hydrochloride (Sigma-Aldrich) for 45’, 100 nM Wortmannin (Sigma-Aldrich) for 30’, and 100 U/ml Nystatin for 15’. At the end of incubation, DiD-LNCs and Blank-LNCs were added as previously described. After 3h (PBLs) or overnight (tumor cell suspension) incubation, cells were washed twice with culture medium and stained for flow cytometry analysis.

**Multiparametric flow cytometry**

For flow cytometry staining, cells were washed with Dulbecco’s phosphate buffered saline (PBS, LONZA) plus 1% FBS and incubated with Fc-Receptor Blocking reagent (FcR, Miltenyi Biotec) at 4°C for 10’. Then fluorochrome-conjugated mAbs were added for 20’ at 4°C. At the

end of incubation, cells were washed and resuspended in PBS plus 1% FBS for flow cytometry acquisition.

Leukocyte subsets were identified by staining with anti-CD14 FITC (Miltenyi Biotec), anti-CD15 V450 (BD Biosciences), anti-IL4Rα PE (R&D Systems), anti-HLA-DR PerCP Cy5.5 (Biolegend), anti-CD16 FITC (Miltenyi Biotec), anti-CD11b PE (Beckman Coulter), anti-CD3 PE-Cy7 (Beckman Coulter), anti-CD19 FITC (BD Biosciences), and anti-CD56 FITC (BD Biosciences).

Tumor cell suspension was stained with LIVE/DEAD Fixable Aqua (Life Techonologies), anti- CD45 BV421 (BD Biosciences), anti-CD14 FITC (Miltenyi Biotec), anti-HLA-DR PerCP-Cy5.5 (Biolegend), and anti-CD49D PE (BioLegend).

Data acquisition was performed using LSRII flow cytometer (BD Biosciences) equipped with 4 lasers (405nm, 488nm, 561nm, 640nm), and results were analyzed by FlowJo software (Three Star Inc).

Fluorescence minus one (FMO) controls for HLA-DR and IL4Rα were used as negative controls. All antibodies used for flow cytometry were titrated in a lot-dependent manner.

**Confocal microscopy**

PBMCs and PMNs were incubated with neutral 100 nm LNCs and nystatin inhibitor as specified for flow cytometry analysis. Afterwards, 5*10^5^ PBMCs and PMNs were seeded on coverslips for 2 hours in 24-well plates and washed three times with PBS to eliminate non-adhering cells, fixed with 4% paraformaldehyde (Sigma-Aldrich) for 10’ at RT, and blocked with PBS 0,05% Triton plus 20% NGS (normal goat serum, Vector Laboratories, Burlingame, CA, USA) for 2 hours at RT. Nuclei were counterstained with DAPI (Sigma-Aldrich). Samples were analyzed under a laser scanning confocal microscope (Leica TCS SP5, Wetzlar, Germany) equipped with 4 lasers (405nm/ Argon-458,476,488,494,514nm-/ 561nm/ 633nm), and results were analyzed by Las X (Leica MICROSYSTEMS).

**Statistical analysis**

The Mann-Whitney and the Student t-test were used as appropriate to evaluate statistically significant variations between groups of samples. All tests were two-sided and a P < 0.05 was considered statistically significant. Absence of significance was not reported for brevity. Statistical analyses were performed using the Sigmaplot software (Systat Software Inc., CA, USA).
